# Genetic and Functional Diversity of *Pseudomonas aeruginosa* in Patients With Chronic Obstructive Pulmonary Disease

**DOI:** 10.3389/fmicb.2020.598478

**Published:** 2020-10-29

**Authors:** Kelei Zhao, Ting Huang, Jiafu Lin, Chaochao Yan, Lianming Du, Tao Song, Jing Li, Yidong Guo, Yiwen Chu, Junfeng Deng, Xinrong Wang, Chaolan Liu, Yingshun Zhou

**Affiliations:** ^1^Antibiotics Research and Re-evaluation Key Laboratory of Sichuan Province, Sichuan Industrial Institute of Antibiotics, Chengdu University, Chengdu, China; ^2^Ecological Restoration and Biodiversity Conservation Key Laboratory of Sichuan Province, Chengdu Institute of Biology, Chinese Academy of Sciences, Chengdu, China; ^3^Department of Pathogenic Biology, College of Preclinical Medicine, Southwest Medical University, Luzhou, China

**Keywords:** chronic obstructive pulmonary disease, *Pseudomonas aeruginosa*, whole-genome sequencing, phylogeny, functional profiling, transcriptional pattern

## Abstract

*Pseudomonas aeruginosa* is the most relevant pathogen to the severe exacerbations of patients with chronic obstructive pulmonary disease (COPD). However, the genetic and functional characteristics of *P. aeruginosa* isolates from COPD airways still remain less understood. In this study, the genetic, phylogenetic, phenotypic, and transcriptional features of *P. aeruginosa* isolates from COPD sputa were comprehensively explored by susceptibility testing, comparative-genomic analysis, phylogenetic analysis, phenotypic profiling, and comparative-transcriptomic analysis. We found that *P. aeruginosa* was prevalent in elder COPD patients and highly resisted to many commonly used antibiotics. *P. aeruginosa* COPD isolates harbored a substantial number of variant sites that might influence the primary metabolism and substance transport system. These isolates were discretely distributed in the phylogenetic tree and clustered with internationally collected *P. aeruginosa* in two major groups, and could be classified into three groups according to their differences in virulence-related phenotypes. Furthermore, the transcriptional patterns of COPD isolates could be classified into PAO1-like group with reduced protein secretion and motility and PAO1-distinct group with decreased substance transport but enhanced primary metabolism. In conclusion, this study demonstrates that *P. aeruginosa* isolates from COPD patients have abundant genetic and phenotypic diversity, and provides an important reference for further exploring the survival strategy of *P. aeruginosa* in COPD airways and the development of anti-pseudomonal therapy.

## Introduction

As a common public health problem, the increasing prevalence, morbidity, and mortality of chronic obstructive pulmonary disease (COPD) cause major healthcare and economic burden worldwide, and bring a huge challenge for scientists and clinicians within the twenty-first century (López-Campos et al., 2016; Rabe and Watz, 2017). From 1990 to 2015, the global prevalence of COPD increased by approximately 44.2% to 174.5 million individuals with 3.2 million people dying from this disease in 2015, and COPD remains the most prevalent chronic respiratory disease by 2017. It is projected that COPD causes 5% of all deaths worldwide and will be the third leading cause of death (4.7 million individuals) in 2020 (Murray and Lopez, 1997; GBD 2013 Mortality and Causes of Death Collaborators, 2015; GBD 2015 Chronic Respiratory Disease Collaborators, 2017; GBD Chronic Respiratory Disease Collaborators, 2020).

Different from the genetic disease cystic fibrosis (CF) caused by the mutations in the CF transmembrane conductance regulator, it is well recognized that tobacco smoking is the main causal and primary inciter for the formation of COPD (Rab et al., 2013; Lange et al., 2015; De Rose et al., 2018). Because of the limitation of lung function, COPD patients normally have common clinical symptoms like dyspnea, cough, excessive phlegm, wheezing, at high risk of developing several comorbidities, and are susceptible to colonization by multiple pathogenic bacterial species (Negewo et al., 2015; Poh et al., 2017; Budden et al., 2019). The severe exacerbations of COPD are generally associated with respiratory infections, which have a major impact on the quality of life and are the main cause of death among COPD patients (Dickson et al., 2014; Cavallazzi and Ramirez, 2020).

*Pseudomonas aeruginosa* is a versatile opportunistic pathogen capable of colonizing a wide range of hosts, and considered to be the most relevant pathogen to the severe exacerbations and deaths of COPD patients (Garcia-Vidal et al., 2009; Cullen and McClean, 2015). This microorganism has a relatively large genome size and flexible intracellular regulation that contribute to the prosperity of population in different habitats and infection conditions, including acute infection and chronic persistence (Stover et al., 2000; Balasubramanian et al., 2013; Moradali et al., 2017; Zhao et al., 2019). As a predominant bacterial species in CF patients, the adaptative and pathogenic mechanisms of *P. aeruginosa* CF isolates have been well characterized (Smith et al., 2006; Yang et al., 2011; Yonker et al., 2015; Moradali et al., 2017). By contrast, the genetic diversity and functional pattern of *P. aeruginosa* isolates from COPD airways are less studied.

Evolution of *P. aeruginosa* in chronic airway infections frequently results in loss of O-antigen and cell motility, increased antibiotic resistance, mucoid production, and mutation rate, but decreased quorum-sensing (QS) regulation and virulence (Smith et al., 2006; Martínez-Solano et al., 2008; Rakhimova et al., 2009; Yang et al., 2011; Jiricny et al., 2014; Cullen and McClean, 2015). Despite the different etiology and pathogenesis between CF and COPD, these two diseases have many overlaps in the key phenotypical, pathologic, and clinical features in airways (Decramer et al., 2012; Elborn, 2016; De Rose et al., 2018; Fernandez et al., 2018). However, in comparison with the earlier colonization and pervasive persistence of *P. aeruginosa* in CF airways, isolation of *P. aeruginosa* from COPD is more frequent in hospitalized patients with severe exacerbations (Planquette et al., 2015; Yonker et al., 2015; Rodrigo-Troyano et al., 2018; Cavallazzi and Ramirez, 2020; Eklöf et al., 2020). This difference indicates that survival of *P. aeruginosa* in COPD airways might have relatively less time to adapt to the lung environment, and thus might have abundant genetic and functional diversity.

In this study, we first checked the prevalence of *P. aeruginosa* in the sputum samples of hospitalized COPD patients. By using *P. aeruginosa* PAO1 as the reference strain, the genetic and phenotypic features of *P. aeruginosa* COPD isolates were then comprehensively identified by antibiotic resistance profiling, whole-genome sequencing, phylogenetic analysis, phenotypic profiling, and transcriptional profiling in a stepwise manner. Finally, we showed that the *P. aeruginosa* COPD isolates could be generally divided into two or three groups with abundant genetic and phenotypic diversity. Moreover, reduced substance transport and cell motility but enhanced primary metabolisms might contribute to the colonization of *P. aeruginosa* in COPD airways.

## Materials and Methods

### Ethical Statement

Sputum samples were obtained from the COPD patients hospitalized in the affiliated hospital of Southwest Medical University (Sichuan, China). Written informed consents were received from the patients or the patients’ immediate family members. The study was approved by the Southwest Medical University Ethics Committee, and all methods were carried out in accordance with the guidelines and regulations of Southwest Medical University.

### Sputum Samples and Identification of *P. aeruginosa*

During 2017–2018, 1,605 morning sputum samples were collected from 526 COPD patients (spontaneously expectorated) and 9 newborns (swabbed by sterile cotton swab in the throat) with acute pneumonia hospitalized in the affiliated hospital of Southwest Medical University. Three sputum samples were obtained from each patient in 3 days (one sputum per day), and immediately spread on cetyltrimethylammonium bromide and King’s B plates for the selection of *P. aeruginosa*, followed by 16S rRNA identification. Wild-type *P. aeruginosa* PAO1 was previously preserved in the laboratory and described elsewhere (Zhao et al., 2019). All the *P. aeruginosa* isolates were routinely cultured in lysogeny broth (LB) from a single colony at 37°C with shaking (220 rpm).

### Susceptibility Testing

Susceptibilities of *P. aeruginosa* isolates to ciprofloxacin, levofloxacin, gentamicin, imipenem, amikacin, tobramycin, cefepime, ampicillin, ampicillin/sulbactam (Amp/sulbactam), piperacillin/tazobactam (Pip/Tazo), ceftazidime, cefazolin, cefotetan, ceftriaxone, nitrofurantoin, and trimethoprim/sulfamethoxazole (Trimeth/Sulfa) were determined by plate dilution method according to the guidelines of (Clinical and Laboratory Standards Institute CLSI, 2015). Minimal inhibitory concentration (MIC) values–based heatmap with correlation analysis was generated by using HemI (Deng et al., 2014).

### Whole-Genome Sequencing

Genomic DNAs of *P. aeruginosa* isolates were harvested and conducted for library construction using NEBNext Ultra DNA Library Prep Kit for Illumina (New England Biolabs, United States), followed by whole-genome sequencing on the Illumina HiSeq PE150 platform (Novogene Bioinformatics Technology, China). The sequencing data are deposited in the NCBI BioProject database under accession number PRJNA650511. The filtered reads were mapped to the genome sequence of PAO1 (GenBank accession number AE004091) using BWA-MEM algorithm within BWA v0.7.8 (Li and Durbin, 2009) and Picard v2.18.17^1^. SAMtools v0.1.18 was used to detect the single-nucleotide polymorphism (SNP) sites and insertion/deletion (InDel) sites (Li et al., 2009), and GATK v4.0.11.0 IndelRealigner was then used to exclude the false-positive SNPs by local realignment (McKenna et al., 2010). The mutation impact of these SNPs was evaluated by SnpEff v4.3 (Cingolani et al., 2012). Kyoto Encyclopedia of Genes and Genomes (KEGG) pathway prediction and protein classification were performed by combined use of KOBAS v2.0 (Mao et al., 2005) and DAVID Bioinformatics Resources^2^. Mutations that happened in virulence genes were determined by mapping the genes with SNP or InDel mutations to the VFDB database^3^ (Liu et al., 2019). Venn diagram was used to get the common and different numbers of SNPs, InDels, genes, and their enriched functional categories^4^.

### Phylogenetic Analysis

High-quality pair-end reads generated from the aforementioned whole-genome sequencing were used to *de novo* assemble genomes for the 22 *P. aeruginosa* clinical isolates with Platanus v1.2.4 (Kajitani et al., 2014). The program ran with 4 threads and a limited 80 G memory. The initial k-mer size was set to 31, and the step size of k-mer extension was set to 10. Other parameters of assembly were left as default. Subsequently, the assembled contigs of *P. aeruginosa* in this study, and the completed genome sequences of 46 internationally collected *P. aeruginosa* isolates and 1 outgroup species *Azotobacter vinelandii* retrieved from the NCBI database (Supplementary Table 1), were used to construct a phylogenetic tree with kSNPs v3.1 (Gardner et al., 2015). The k-mer was set to 19, and the tree was visualized with FigTree v1.4.3^5^ and iTOL v4 (Letunic and Bork, 2019).

### Phenotypic Identification

Overnight cultured *P. aeruginosa* isolates were adjusted to optical density (OD_600_) of 1.0 in sterile saline as ready-to-use solutions. Phenotypes of *P. aeruginosa* isolates were identified according to the protocols edited by Filloux and Ramos (2014). Briefly, LasR function and production of extracellular proteases were evaluated by inoculating 5 μl of *P. aeruginosa* solution on adenosine (0.1%) plates to check the growth status of colony, and on skim-milk (0.5%) plates to measure the size of proteolytic halo around the colony. For pyocyanin production, 10 μl of *P. aeruginosa* solution was inoculated in 4 ml of LB medium and cultured overnight. After measuring the cell density at OD_600_, pyocyanin was extracted from the supernatant by chloroform and HCl, followed by absorbance measurement at 520 nm. For biofilm production, 10 μl of *P. aeruginosa* solution was inoculated in a sterile 96-well plate containing 200 μl of LB medium in the well and statically cultured overnight. After measuring the cell density at OD_600_, the culture liquid was gently removed and the yield of biofilm was determined by crystal violet staining, dissolution, and then measuring the OD_595_ value. Furthermore, bacterial cells were inoculated or stabbed on modified M8 plates containing 0.3 and 1.0% agar powder and statically cultured for 24 h, and then the swimming and twitching motilities were determined by measuring the diameters of colonies. Each experiment was independently repeated three times, and the values of *P. aeruginosa* COPD isolates were compared with those of PAO1. The web tool ClustVis^6^ was used to visualize the phenotypic profiling data in heatmap by setting a significantly enhanced phenotype as “1,” decreased as “−1,” and no significant change as “0” (Metsalu and Vilo, 2015).

### *Caenorhabditis elegans* Killing Assays

*C. elegans* was used as a high-throughput method to determine the pathogenicity of *P. aeruginosa* isolates identified in this study. A total of 100 μl of each *P. aeruginosa* solution was spread on 3.5 cm-diameter peptone–glucose–sorbitol plates to mimic acute infection by the fast-killing ability of *P. aeruginosa*, and on nematode growth medium to mimic chronic infection by the slow-killing ability of *P. aeruginosa*, and incubated at 37°C for 24 h (Tan et al., 1999). Subsequently, 10 newly cultured adult nematodes (L4 stage) were seeded on each plate and further incubated at 25°C to monitor the survival status of nematodes. Nematodes fed with *Escherichia coli* OP50 (uracil auxotrophy) or *P. aeruginosa* PAO1 and cultured under the same culture conditions were set as controls.

### Transcriptomic Analysis

*P. aeruginosa* isolates cultured in LB medium were harvested (1 ml) when the cell densities reached OD_600_ = 1.5 (stationary phase), and then the RNAs were isolated using Total RNA Isolation Kit with gDNA removal (Foregene Biotechnology, Co., Ltd., China). Qualified RNA samples from two parallel experiments with three independent biological replicates were conducted for library construction using NEBNext Ultra RNA Library Prep Kit for Illumina. High-quality library was conducted for prokaryotic strand-specific RNA-sequencing (RNA-seq) on the Illumina HiSeq PE150 platform. The sequencing data are deposited in the NCBI BioProject database under accession number PRJNA650511. After mapping the filtered reads to *P. aeruginosa* PAO1 by Bowtie 2 v2.2.3 (Langmead and Salzberg, 2012), HTSeq v0.6.1 (Anders et al., 2015), and DESeq 2 (Love et al., 2014) were used to calculate the values of differential gene expression using expected fragments per kilobase of transcript per million fragments (FPKM). The resulting *p*-values by DESeq were adjusted using the Benjamini and Hochberg’s approach, and genes with an adjusted *p* value (*p*_adj_) < 0.05 was thought to be significantly different. KEGG pathway prediction, Gene Ontology (GO) enrichment analysis, and protein classification were performed by combined application of KOBAS v2.0, GOseq R package, DAVID, and Venn diagram. Differentially expressed QS-regulated genes in *P. aeruginosa* were screened by mapping the genes to previously established list of QS-induced genes (Schuster et al., 2003).

### Statistical Analysis

GraphPad Prism v8.0 (San Diego, CA, United States) was used to process the data generated by the phenotypic identification assays. Mean values of SD were compared by using two-tailed unpaired *t*-test. The survival curves of *C. elegans* were compared by using log-rank (Mantel–Cox) test.

## Results

### Epidemiology and Antibiotic Resistance of *P. aeruginosa* COPD Isolates

**FIGURE 1:**
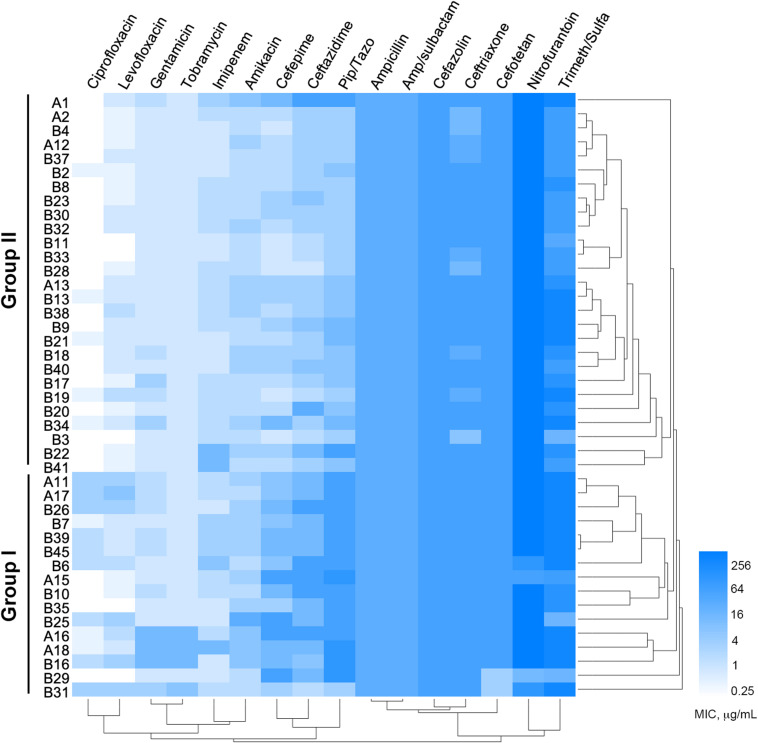
Minimum inhibitory concentrations (MICs) of 16 commonly used antibiotics on *Pseudomonas aeruginosa* isolates from COPD patients. Bar, MIC values (μg/ml).

Among the 1,605 sputum samples from 526 COPD patients, 42 COPD individuals (62.19 ± 15.81 years old, isolation rate ≈ 7.98%) with pneumonia, dyspnea, bronchiectasis, and cough, and one newborn (*n* = 9) with acute pneumonia, were found to be *P. aeruginosa* positive (Supplementary Table 2). All the 43 *P. aeruginosa* isolates (one isolate per patient) were conducted for susceptibility testing and the results showed that (Figure 1) the majority of these isolates were susceptible to imipenem, quinolone, and aminoglycoside antibiotics, but highly resistant to nitrofurantoin, Trimeth/Sulfa, and cephalosporin antibiotics. Notably, four isolates (B7, B30, B33, and B37) from the younger patients (about 30 years of age) and one (A2) from the newborn were susceptible to at least 8 of the 16 tested antibiotics. MIC value–based correlation analysis revealed that these *P. aeruginosa* isolates could be classified into two main groups with several sub-groups. A total of 21 COPD isolates (19 from elder patients and 2 from younger), which were allocated in the main branch or randomly selected from each sub-group, were chosen to further characterize their genetic and functional features. Moreover, *P. aeruginosa* isolate A2, which was isolated from the newborn patient who experienced less antibiotic treatment, was included as potentially less evolved isolate along with *P. aeruginosa* PAO1.

### *P. aeruginosa* COPD Isolates Have Remarkable Genetic Diversity

By using *P. aeruginosa* PAO1 as the common reference, the results of whole-genome sequencing revealed that the map rates of COPD isolates ranged from 79.01 to 94.81% (Supplementary Table 3). A large number of SNPs (ranging from 26,908 to 53,443) including 5,161–9,429 non-synonymous sites in 2,454–3,355 genes, and InDels (ranging from 417 to 741) in 95–149 genes including 38–64 shift sites were identified (Supplementary Table 4 and Supplementary Datasets 1, 2). SNP effect analysis suggested that the transitions/transversions ratio of SNPs in *P. aeruginosa* COPD isolates was approximately 3.2. The overwhelming majority of SNPs were silent mutation and had modifier effect on gene function, whereas the numbers of high-impact and nonsense SNPs only ranged from 8 to 30 and 5–17 (Supplementary Dataset 3).

To test whether the remarkable genotypic differences between *P. aeruginosa* PAO1 and the COPD isolates identified in this study were caused by the endemicity of clinical isolates, the COPD isolate B29, which had the fewest variant genes compared with PAO1, was set as internal reference and compared with other isolates. We found that the number of genes with non-synonymous SNP and InDel sites in other 21 isolates ranged from 29 to 1,314 and 4–88, respectively. Moreover, although these isolates could be classified into three distinct groups according to the number of variant genes (uniquely), there were no apparent classification rules to follow based on the known antibiotic resistance pattern and infection status (Supplementary Table 5). Therefore, these results demonstrated the genetic diversity of *P. aeruginosa* COPD isolates, which had some genetic similarities among them but remarkably distinct from the reference strain PAO1.

### Phylogenetic Relationship of *P. aeruginosa* COPD Isolates With Other Sources

**FIGURE 2:**
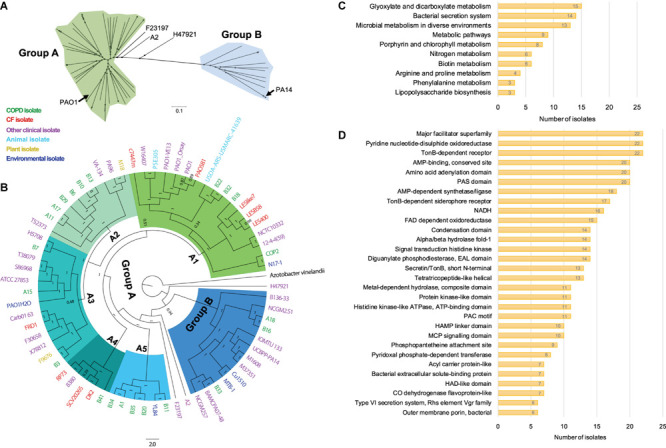
Phylogenetic analysis and functional enrichment of genes with non-synonymous SNPs among *P. aeruginosa* COPD isolates. **(A)** Unrooted tree showing the phylogeny of all *P. aeruginosa* isolates tested in this study. **(B)** Phylogenetic tree of *P. aeruginosa* isolates identified in this study and other 46 internationally collected isolates constructed based on the SNPs (compared with PAO1) using maximum-likelihood method. *Azotobacter vinelandii* was set as outgroup. Numbers on the nodes indicate bootstrap values for branches. The names of *P. aeruginosa* isolates are colored according to the sources. Green, COPD isolate. Red, CF isolate. Purple, other clinical isolate. Aqua, animal isolate. Yellow, plant isolate. Mazarine blue, environmental isolate. The region of group B is colored in dodger blue, and group A is composed of the remaining five sub-groups (A1–A5). **(C)** Number of *P. aeruginosa* COPD isolates harboring the commonly enriched KEGG terms by the genes with non-synonymous SNPs (*p* < 0.05). **(D)** Number of *P. aeruginosa* COPD isolates harboring the commonly enriched protein classification terms by the genes with non-synonymous SNPs (*p* < 0.05).

To probe the phylogenetic relationship of currently identified *P. aeruginosa* isolates with other internationally collected isolates (Supplementary Table 1), a total of 510,245 SNP loci were called from the genome sequences of 68 *P. aeruginosa* isolates and the outgroup species *Azotobacter vinelandii* by setting *P. aeruginosa* PAO1 as reference, and then conducted for phylogenetic analysis using the maximum-likelihood method. We found that all the tested *P. aeruginosa* isolates could be divided into two main groups with PAO1 in the larger group (group A), whereas PA14 was located in group B (Figure 2A). The *P. aeruginosa* COPD isolates distributed discretely in the phylogenetic tree and showed no apparent geographical specificity or habitat preference (Figure 2B).

Group A was composed of five sub-groups with different support rates (ranging from 0.51 to 1) for the classification, indicating the unstable phylogenetic relationship between some sub-groups (Figure 2B). Specifically, isolate B18 was credibly clustered with the Liverpool epidemic CF strains, whereas B22 and B32 were adjacent to the PAO1 lineage. These sub-branches formed an unstable branch but credibly allocated with the environmental isolate N17-1 and the COPD isolate COP2 under the sub-group A1. Sub-group A2 was composed of six COPD isolates (A11, A17, B6, B10, B13, and B29) with the fewest variant genes, the plant isolate M18, and the clinical isolates PA96 and VA-134 (Figure 2B and Supplementary Table 5). Sub-group A3 was composed of three COPD isolates (A15, B3, and B7) and isolates from different sources. Isolates A1, B11, B20, B34, and B35 were clustered with the environmental isolates YL84 in sub-group A5, whereas B41 was clustered with the epidemic CF isolate DK2 in sub-group A4. Moreover, isolates A18, B16, and B33 with abundant variant genes were clustered with eight other clinical isolates (including PA14) and two environmental isolates in group B, and this group was credibly distributed in the position closing to the root of phylogenetic tree (Figure 2B and Supplementary Table 5). Therefore, our findings here further confirmed the genetic diversity of *P. aeruginosa* isolates from COPD airways and highlighted the formidable cross-colonization capacity of this microorganism.

### Functional Enrichment of Variant Genes Among *P. aeruginosa* COPD Isolates

We then explored the functional enrichment of variant genes in COPD isolates to predict the key functional terms that might be associated with the colonization of *P. aeruginosa* in COPD airways. Compared with PAO1, more than half of the COPD isolates harbored non-synonymous SNPs in the genes significantly enriched (*p* < 0.05) in the KEGG terms of glyoxylate and dicarboxylate metabolism (15 isolates), bacterial secretion system (14 isolates), and microbial metabolism in diverse environments (13 isolates) (Figure 2C and Supplementary Dataset 4). In addition, enrichment of variant genes encoding the proteins belonging to the major facilitator superfamily, pyridine nucleotide-disulfide oxidoreductase, and TonB-dependent receptor were detected in all the 22 isolates (Figure 2D and Supplementary Dataset 4). For the genes harboring at least one shift InDel site, bacterial secretion system was the sole KEGG term that was significantly enriched (*p* < 0.05) in each isolate, and 12 isolates harbored the variant genes encoding the proteins belonging to the major facilitator superfamily (Supplementary Dataset 5).

After checking the variant sites in specific genes, we found that in contrast to the rarely distributed high-impact SNP sites, shift InDels were prevalent in the core genes related to the H2-T6SS (Type 6 Secretion System), T3SS, and membrane transporters (Supplementary Table 6). By applying the variant genes to the virulence factor database of *P. aeruginosa*, 37 virulence factor–encoding genes harboring abundant SNPs and InDels were identified (Table 1). Although high-impact SNP sites happened rarely in these virulence genes, more than half of the COPD isolates carried at least one InDel site in the T6SS protein Fha1, chemotactic signal transduction system protein ChpA, T3SS translocation protein PscP, T3SS regulatory protein PcrH, T3SS export protein PscK, esterase EstA, alginate regulatory protein AlgP, and transporter TonB1. Therefore, we summarized that dysregulated substance transport might contribute to the survival of *P. aeruginosa* in COPD airways.

**TABLE 1:** Variant sites in 37 virulence genes of 22 *P. aeruginosa* clinical isolates compared with the reference genome PAO1.

			**Number of variant sites**			**Number of strains**		
**Name**	**Gene ID**	**Description**	**Synonymous SNPs (types)**	**Non-synonymous SNPs (types)**	**InDels (types)**	**High-impact SNPs**	**InDels**	**Total**
*PA0041*	PA0041	Hemagglutinin	984 (196)	320 (72)	2 (1)	0	2	2
*ppkA*	PA0074	Serine/threonine protein kinase	329 (44)	69 (12)	1 (1)	0	1	1
*icmF1*	PA0077	Type VI secretion protein	281 (55)	40 (6)	9 (2)	0	9	9
*fha1*	PA0081	Fha domain-containing protein	30 (18)	17 (10)	21 (16)	0	21	21
*pilJ*	PA0411	Twitching motility protein	106 (18)	18 (1)	2 (2)	0	2	2
*chpA*	PA0413	Chemotactic signal transduction system Protein	534 (122)	178 (45)	15 (3)	0	12	12
*mucA*	PA0763	Sigma factor AlgU negative regulator	48 (9)	3 (2)	1 (1)	0	1	1
*mucB*	PA0764	Sigma factor AlgU regulator	41 (14)	5 (3)	1 (1)	0	1	1
*gacS*	PA0928	Sensor/response regulator hybrid protein	222 (50)	6 (4)	2 (1)	0	2	2
*fleR*	PA1099	Two-component response regulator	149 (30)	13 (8)	2 (1)	0	2	2
*toxA*	PA1148	Exotoxin A	178 (33)	71 (23)	7 (2)	0	7	7
*lasR*	PA1430	Transcriptional regulator	17 (6)	2 (2)	2 (2)	0	2	2
*pscP*	PA1695	Translocation protein in type III secretion	88 (18)	174 (28)	33 (11)	0	19	19
*pcrH*	PA1707	Regulatory protein PcrH	23 (5)	18 (2)	22 (1)	0	22	22
*pscK*	PA1724	Type III export protein	72 (20)	9 (2)	22 (1)	0	22	22
*exoY*	PA2191	Adenylate cyclase	66 (18)	53 (13)	2 (1)	0	2	2
*PA3142*	PA2402	Peptide synthase	1,728(276)	426 (100)	1 (1)	0	1	1
*pvdL*	PA2424	Peptide synthase	1,746(330)	440 (91)	1 (1)	0	1	1
*PA3142*	PA2573	Chemotaxis transducer	371 (56)	54 (11)	2 (1)	0	2	2
*fimV*	PA3115	Motility protein	209 (46)	36 (12)	7 (3)	1	5	6
*PA3142*	PA3142	Hypothetical protein	8 (3)	21 (7)	5 (3)	0	5	5
*rhlR*	PA3477	Transcriptional regulator	83 (12)	0	1 (1)	0	1	1
*algI*	PA3548	Alginate o-acetylase	46 (13)	71 (8)	0	1	0	1
*pchF*	PA4225	Pyochelin synthetase	558 (93)	279 (62)	6 (2)	0	6	6
*pchE*	PA4226	Dihydroaeruginoic acid synthetase	384 (60)	208 (43)	2 (2)	0	2	2
*pchA*	PA4231	Salicylate biosynthesis isochorismate synthase	134 (22)	32 (10)	1 (1)	0	1	1
*pilA*	PA4525	Type 4 fimbrial protein	26 (11)	0	1 (1)	0	1	1
*pilB*	PA4526	Type 4 fimbrial biogenesis protein	957 (148)	78 (17)	1 (1)	0	1	1
*pilY1*	PA4554	Type 4 fimbrial biogenesis protein	901 (167)	186 (45)	1 (1)	0	1	1
*motB*	PA4953	Flagellar motor protein	118 (16)	1 (1)	1 (1)	0	1	1
*pilQ*	PA5040	Type 4 fimbrial biogenesis outer membrane	886 (138)	134 (23)	1 (1)	1	1	2
*pilO*	PA5042	Type 4 fimbrial biogenesis protein	190 (31)	41 (6)	2 (1)	0	2	2
*estA*	PA5112	Esterase	344 (35)	19 (10)	17 (1)	0	17	17
*algP*	PA5253	Alginate regulatory protein	112 (29)	72 (18)	12 (11)	0	10	10
*cyaA*	PA5272	Adenylate cyclase	294 (51)	83 (12)	1 (1)	0	1	1
*PA5441*	PA5441	Hypothetical protein	81 (27)	88 (16)	0	1	0	1
*tonB1*	PA5531	Transporter TonB	302 (32)	2 (2)	18 (1)	1	18	19

### Phenotypic Profiling of *P. aeruginosa* COPD Isolates

**FIGURE 3:**
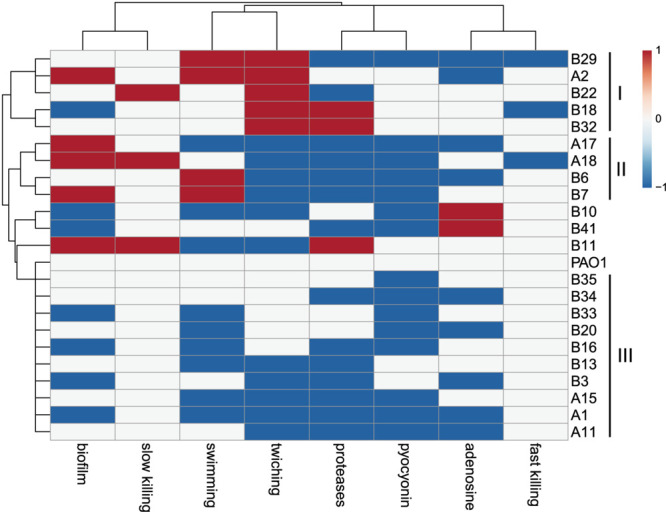
Phenotypic profiling of *P. aeruginosa* COPD isolates compared with PAO1. Heatmap was generated by setting a significantly (*p* < 0.05) enhanced phenotype as “1” (red), decreased as “−1” (blue), and no significant change as “0” (white).

The virulence-related phenotypes of *P. aeruginosa* COPD isolates were profiled to determine the phenotypic diversity of them by using PAO1 as reference. We found that the phenotypes of COPD isolates could be classified into three groups: (I) enhanced cell motility (*n* = 5), (II) enhanced biofilm formation but decreased twitching and extracellular products production (*n* = 4), and (III) decreased biofilm formation, cell motility, and extracellular products production (*n* = 10). Only five COPD isolates showed significant differences in the fast/slow killing of *C. elegans* (Figure 3). These results revealed that the *P. aeruginosa* COPD isolates identified in this study had abundant phenotypic diversity.

### Transcriptional Similarity Among *P. aeruginosa* COPD Isolates

**FIGURE 4:**
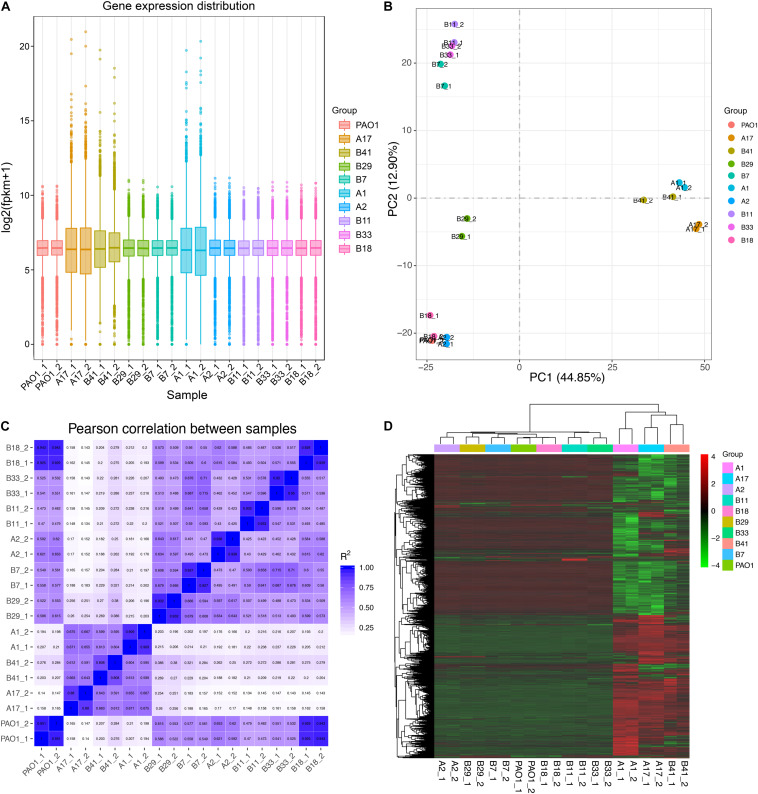
General transcriptional patterns of *P. aeruginosa* COPD isolates based on RNA-seq-determined gene expression values (FPKM). **(A)** Distribution of gene expression in each *P. aeruginosa* isolate and their independent replicates. **(B)** Transcriptional pattern-based relationship among *P. aeruginosa* COPD isolates as determined by PCA. **(C)** Transcriptional pattern-based Pearson correlation between *P. aeruginosa* COPD isolates. **(D)** Single gene expression level-based general correlation among *P. aeruginosa* COPD isolates.

Based on the results of phylogenetic analysis and phenotypic profiling mentioned previously, nine *P. aeruginosa* isolates located in the main branch or randomly selected from each sub-group were chosen to determine their general (stationary phase growth) transcriptional patterns. The results of RNA-seq showed that the gene expression distribution between two replicates were generally the same, but different among isolates (Figure 4A). Principal component analysis (PCA) revealed that the nine *P. aeruginosa* isolates were clustered into two main groups according to their transcriptional similarities with PAO1 (Figure 4B), and the transcription of isolates A1, A17, and B41 was quite different from the others as confirmed by Pearson correlation analysis (Figure 4C). In addition, A1, A17, and B41 had remarkable transcriptional reprogramming while the remaining six isolates had similar transcriptional pattern to PAO1 (Figure 4D). In comparison with PAO1, 3,757 (1,902 downregulated and 1,855 upregulated), 3,735 (1,867 downregulated and 1,868 upregulated), and 2,358 (976 downregulated and 1,382 upregulated) differentially expressed genes (*p*_adj_ < 0.05) were identified in isolates A1, A17, and B41, respectively. By contrast, no transcriptional difference was detected between B18 and PAO1, while relatively fewer differentially expressed genes (196–336) were detected in the other five isolates (Supplementary Dataset 6). Hence, the transcriptional similarities of COPD isolates were separately analyzed as PAO1-distinct group (A1, A17, and B41) and PAO1-like group (A2, B7, B11, B18, B29, and B33).

**FIGURE 5:**
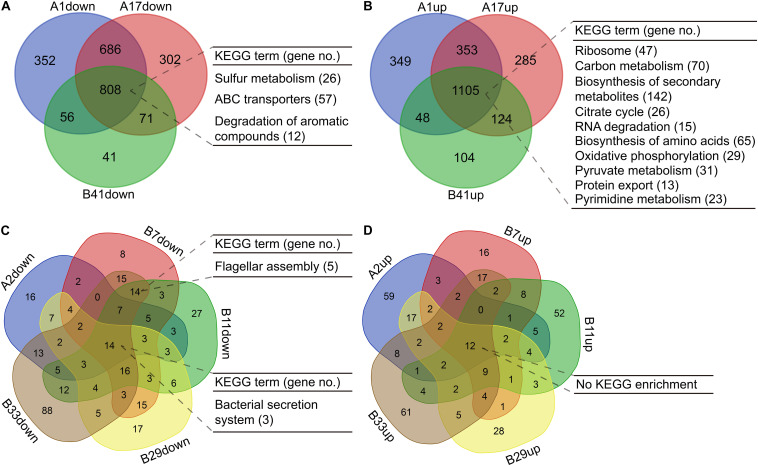
Functional enrichment of differentially expressed genes in *P. aeruginosa* COPD isolates compared with PAO1. **(A)** Significantly enriched KEGG terms by the commonly down-regulated genes in *P. aeruginosa* isolates A1, A17, and B41. **(B)** Significantly enriched KEGG terms by the commonly up-regulated genes in *P. aeruginosa* isolates A1, A17, and B41. **(C)** Significantly enriched KEGG terms by the commonly downregulated genes in *P. aeruginosa* isolates A2, B7, B11, B29, and B33. **(D)** Significantly enriched KEGG terms by the commonly upregulated genes in *P. aeruginosa* isolates A2, B7, B11, B29, and B33. KEGG terms shown are *p* < 0.05, Benjamini < 0.05. All the differentially expressed genes used are *p*_adj_ < 0.05.

For the isolates in PAO1-distinct group, 1,913 commonly differentially expressed genes were obtained, and the commonly downregulated genes (*n* = 808) were significantly (*p* < 0.05, Benjamini < 0.05) enriched in sulfur metabolism, ABC transporters, and degradation of aromatic compounds, whereas the commonly upregulated genes (*n* = 1,105) were enriched in several primary metabolic pathways, biosynthesis of secondary metabolites, protein export, and so on (Figures 5A,B, Supplementary Figures 2, 3 and Supplementary Dataset 7). By contrast, only 26 commonly differentially expressed genes were obtained among the five isolates in PAO1-like group excluding B18, and the commonly downregulated genes (*n* = 14) were enriched in bacterial secretion system. No KEGG term was enriched by the commonly upregulated (*n* = 12) genes. Moreover, flagellar assembly was enriched by the 14 commonly downregulated genes of isolates B7, B11, and B33 (Figures 5C,D, Supplementary Figures 4, 5 and Supplementary Dataset 8). We further profiled the expression of 315 QS-induced genes in both groups and found that the PAO1-like group had only slight transcriptional changes in these genes, while 19 commonly decreased and 38 increased genes were identified in the PAO1-distinct group (Supplementary Figure 6 and Supplementary Dataset 9). Collectively, our data here revealed the transcriptional diversity of *P. aeruginosa* COPD isolates. Decreased substance transport and cell motility but enhanced primary metabolisms might contribute to the colonization of *P. aeruginosa* in COPD airways.

## Discussion

As a common cause of severe exacerbations of COPD, it is estimated that the isolation rates of *P. aeruginosa* ranged from 4 to 18% in COPD sputa (Garcia-Vidal et al., 2009; Rodrigo-Troyano et al., 2018; Eklöf et al., 2020). In this study, 42 *P. aeruginosa* isolates were identified from 526 COPD patients and showed heterogeneous multi-resistance to most of the commonly used antibiotics (Figure 1). Although it was hard to ascertain the time scale of specific *P. aeruginosa* colonization in each patient, the present study reported that *P. aeruginosa* isolates from younger COPD patients might be less resistant and the average age of *P. aeruginosa*–positive COPD patients is 62.19 ± 15.81 (Supplementary Table 2). This was consistent with the previous finding that *P. aeruginosa* isolates were more frequent in elder COPD patients (Garcia-Vidal et al., 2009; Cavallazzi and Ramirez, 2020). Furthermore, our work suggested that resistance pattern, which is associated with the time scale of bacterial colonization and the level of antibiotic selection (Beceiro et al., 2013; Cullen and McClean, 2015; Planquette et al., 2015; Ho and Ip, 2019), could be used to preliminarily predict the phenotypic and genetic diversity of *P. aeruginosa* isolates.

It is generally recognized that the genomes of *P. aeruginosa* isolates from different resources or infection status can be classified into two major phylogenetic groups, namely PAO1-group and PA14-group (Stewart et al., 2014; Freschi et al., 2018, 2019; Ozer et al., 2019). By including the *P. aeruginosa* COPD isolates identified in this study, we found that all the tested *P. aeruginosa* isolates could also be divided into two major groups with high bootstrap values. Differently, group A containing the reference genome PAO1 was more complicated and could further be divided into five sub-groups, and two of the sub-groups were mainly composed by COPD isolates (Figures 2A,B). Moreover, although these COPD isolates were collected from the same hospital in the recent 1–2 years, they were discretely distributed in each clade with no apparent geographical specificity or habitat preference, and thus demonstrated the genetic diversity and cross-colonization capacity of *P. aeruginosa* COPD isolates.

Previous genotyping of *P. aeruginosa* CF isolates suggested that the frequently happened loss-of-function mutations in the central QS regulator LasR, multidrug efflux regulator MexZ, anti-sigma factor MucA, RNA polymerase sigma-54 factor RpoN, and sigma-22 factor AlgT are primarily related to the adaptation and dissemination of *P. aeruginosa* (Martin et al., 1993; Smith et al., 2006; Hoffman et al., 2009; Ciofu et al., 2010; Yang et al., 2011; Feliziani et al., 2014). The work by Martínez-Solano et al. (2008) clearly demonstrated that persistence colonization of *P. aeruginosa* isolates in COPD airways would evolve toward the similar trend of phenotypic change with those in CF. By using PAO1 as reference, we identified a substantial number of variant sites that might influence the substance transport and intracellular metabolism of *P. aeruginosa* COPD isolates (Figures 2C,D and Table 1). The result of phenotypic profiling further showed that the *P. aeruginosa* COPD isolates could be classified into three groups according to their differences in the capacities of cell motilities, biofilm formation, and extracellular products production (Figure 3). In comparison with the previously confirmed reduction of the three capacities of *P. aeruginosa* isolates in chronically colonizing COPD and CF airways (Martínez-Solano et al., 2008; Jiricny et al., 2014; Cullen and McClean, 2015), the phenotypic diversity of COPD isolates identified in our study might be due to their different stages in evolution. We also noticed that the phenotypic profiling–based classification of COPD isolates showed a slight consistency to the clustering of SNP-based phylogenetic tree (Figures 2B, 3). This might be related to the large number of SNPs with modification properties, which had no significant effects on gene functions but could remarkably influence the phylogenetic status.

The results of comparative-transcriptomic analyses further confirmed the functional diversity of *P. aeruginosa* COPD isolates by showing that the intracellular transcription of COPD isolates could be classified into PAO1-distinct group and PAO-like group. In addition, based on phenotypic features of host-adapted *P. aeruginosa* isolates in CF and COPD airways (Martínez-Solano et al., 2008; Jiricny et al., 2014; Cullen and McClean, 2015), our study provided transcriptional evidence that decrease in substance transport and cell motility but increase in primary metabolism might contribute to the persistent colonization of *P. aeruginosa* in COPD patients (Figures 4, 5). Moreover, although the key QS regulatory genes *lasR* and *rhlR* were commonly upregulated in the PAO1-distinct group, the expression of their downstream genes were generally consistent with the phenotypes. By contrast, the comparable expression of QS-induced genes between PAO1-like group and PAO1 was not well matched to their phenotypes (Figure 3, Supplementary Figure 6, and Supplementary Dataset 9). These inconsistencies might be related to the intrinsically complicated intracellular regulatory network of *P. aeruginosa* and the abundant variant sites in substance transport-related genes. Further mechanistic study regarding the intracellular regulation of *P. aeruginosa* might contribute to resolving the inconsistency of gene expression and phenotype in *P. aeruginosa* COPD isolates identified in the present study. Therefore, our study lays an important basis for further exploring the pathogenesis of *P. aeruginosa*-related COPD, and provides references for the development of clinical therapies against this disease.

## Data Availability Statement

The datasets presented in this study can be found in online repositories. The names of the repository/repositories and accession number(s) can be found in the article/ Supplementary Material.

## Ethics Statement

The studies involving human participants were reviewed and approved by the Southwest Medical University Ethics Committee. Written informed consent to participate in this study was provided by the participants’ legal guardian/next of kin.

## Author Contributions

KZ and YZ designed research and coordinated collection of sputum samples. KZ, TH, JLin, TS, JLi, and CL performed experiments. KZ, CY, and LD performed bioinformatic analyses. YG, YC, JD, and XW provided critical experimental equipment and materials. KZ and YZ analyzed data and wrote the manuscript. All authors contributed to the article and approved the submitted version.

## Conflict of Interest

The authors declare that the research was conducted in the absence of any commercial or financial relationships that could be construed as a potential conflict of interest.

## Footnotes

1 http://picard.sourceforge.net

2 https://david.ncifcrf.gov/tools.jsp

3 http://www.mgc.ac.cn/cgi-bin/VFs/genus.cgi?Genus=Pseudomonas

4 http://bioinformatics.psb.ugent.be/webtools/Venn/

5 http://tree.bio.ed.ac.uk/software/figtree/

6 https://biit.cs.ut.ee/clustvis/
