# Supplementary material for: Genetic and Functional Diversity of Pseudomonas aeruginosa in Patients With Chronic Obstructive Pulmonary Disease
Source: Front Microbiol. 2020 Oct 29;11:598478. doi: 10.3389/fmicb.2020.598478 (PMC7673450; doi:10.3389/fmicb.2020.598478)
Supplement: Supplementary file 10 [file Data_Sheet_1.PDF]

Genetic and Functional Diversity of *Pseudomonas aeruginosa* in Patients with Chronic Obstructive Pulmonary Disease

Kelei Zhao[1], Ting Huang[1], Jiafu Lin[1], Chaochao Yan[2], Lianming Du[1], Tao Song[1], Jing Li[1], Yidong Guo[1], Yiwen Chu[1], Junfeng Deng[1], Xinrong Wang[1], Chaolan Liu[1], and Yingshun Zhou[3]

[1] Antibiotics Research and Re-evaluation Key Laboratory of Sichuan Province, Sichuan Industrial Institute of Antibiotics, Chengdu University, Chengdu, Sichuan, China, [2] Ecological Restoration and Biodiversity Conservation Key Laboratory of Sichuan Province, Chengdu Institute of Biology, Chinese Academy of Sciences, Chengdu, Sichuan, China, [3] Department of Pathogenic Biology, College of Preclinical Medicine, Southwest Medical University, Luzhou, Sichuan, China.

Correspondence:

Kelei Zhao, address: No. 168, Huaguan Road, Chengdu 610052, Sichuan, China. Tel.: +86–028–84216035. Email: zhaokelei@cdu.edu.cn.

Yingshun Zhou, address: No. 319, Zhongshan Road, Luzhou 646000, Sichuan, China. Tel.: +86–0830–3160073. Email: yingshunzhou@swmu.edu.cn.

This file contains Supplementary Figures 1-5, Tables 1-6 and legends of Datasets 1-9.

Supplementary Figures

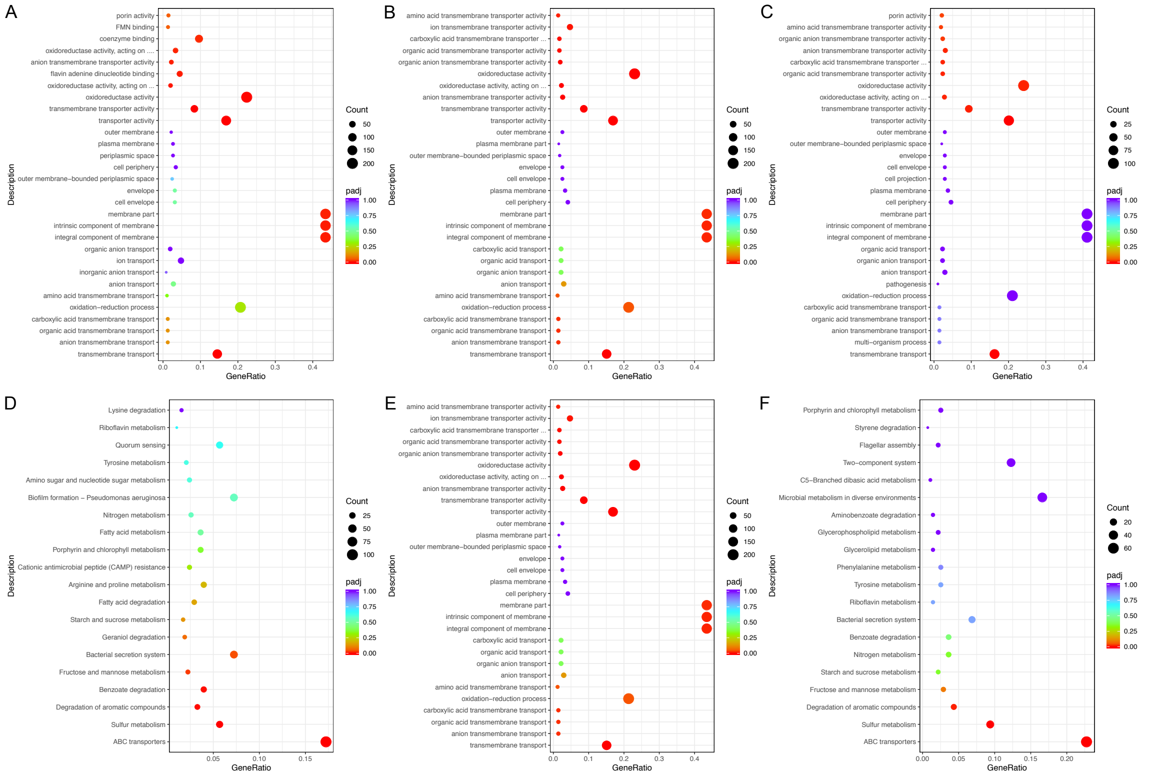

Supplementary Figure 1. Functional enrichment of significantly (padj < 0.05) down-regulated genes in *P. aeruginosa* isolates A1, A17, and B41 compared to the reference strain PAO1. (**A–C**) Enriched GO terms by the significantly down-regulated genes of *P. aeruginosa* isolates A1 **(A)**, A17 **(B),** and B41 **(C)**. (**D–F**) Enriched KEGG terms by the significantly down-regulated genes of *P. aeruginosa* isolates A1 **(D)**, A17 **(E),** and B41 **(F)**.

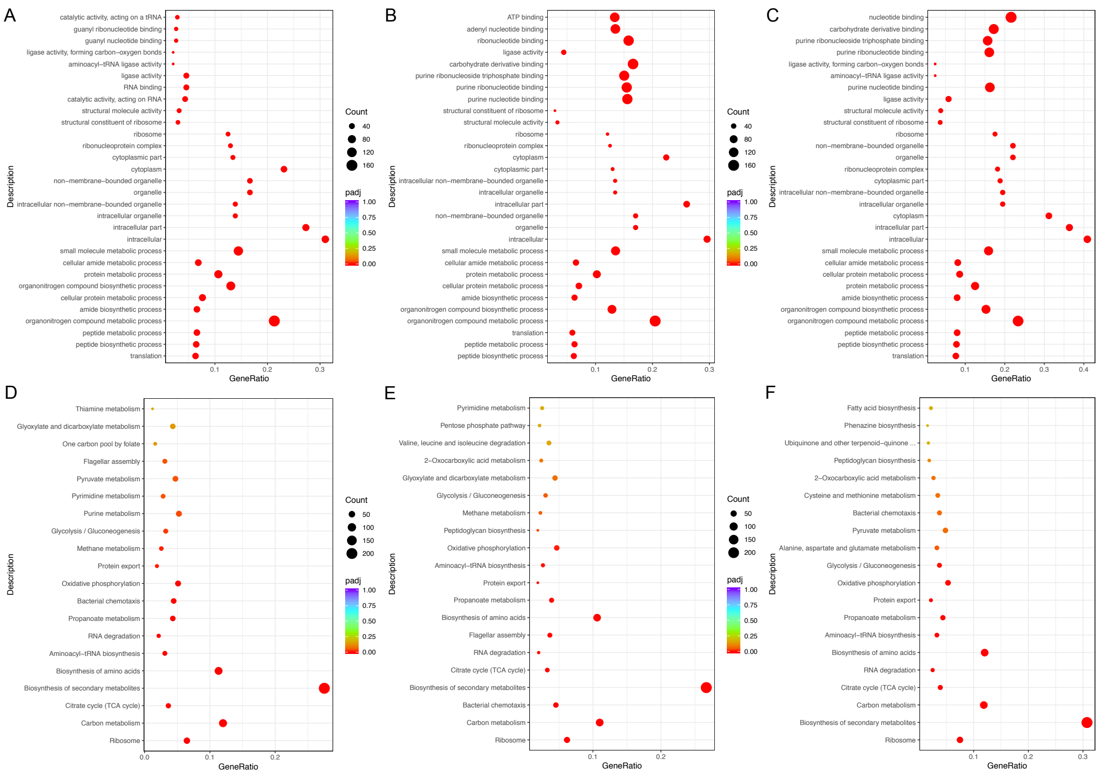

Supplementary Figure 2. Functional enrichment of significantly (padj < 0.05) up-regulated genes in *P. aeruginosa* isolates A1, A17 and B41 compared to the reference strain PAO1. (**A–C)** Enriched GO terms by the significantly up-regulated genes of *P. aeruginosa* isolates A1 **(A)**, A17 **(B),** and B41 **(C)**. (**D–F**) Enriched KEGG terms by the significantly up-regulated genes of *P. aeruginosa* isolates A1 **(D)**, A17 **(E),** and B41 **(F)**.

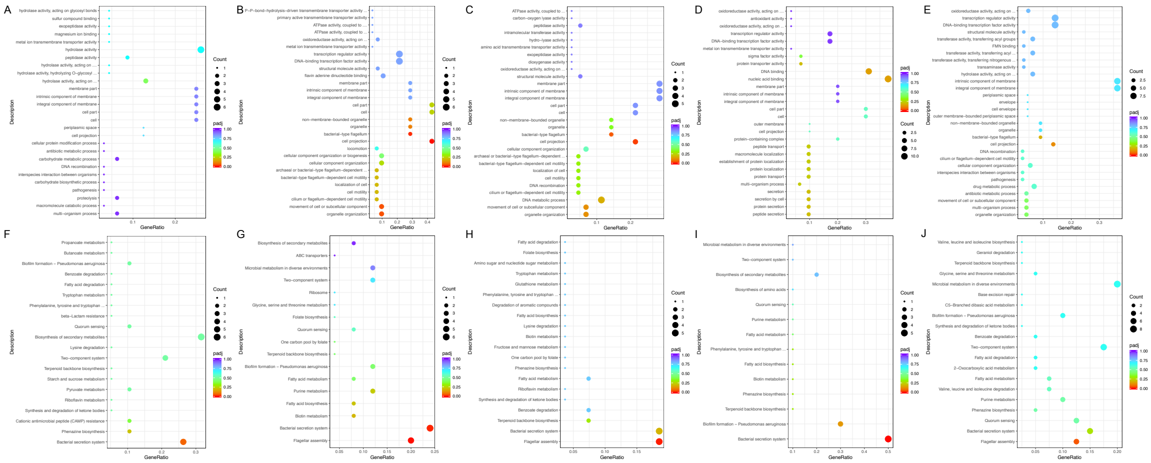

Supplementary Figure 3. Functional enrichment of significantly (padj < 0.05) down-regulated genes in *P. aeruginosa* isolates A2, B7, B11, B29, and B33 compared to the reference strain PAO1. (**A–E)** Enriched GO terms by the significantly down-regulated genes of *P. aeruginosa* isolates A2 **(A)**, B7 **(B)**, B11 **(C)**, B29 **(D),** and B33 **(E)**. **(F–J)** Enriched KEGG terms by the significantly down-regulated genes of *P. aeruginosa* isolates A2 **(F)**, B7 **(G)**, B11 **(H)**, B29 **(I),** and B33 **(J)**.

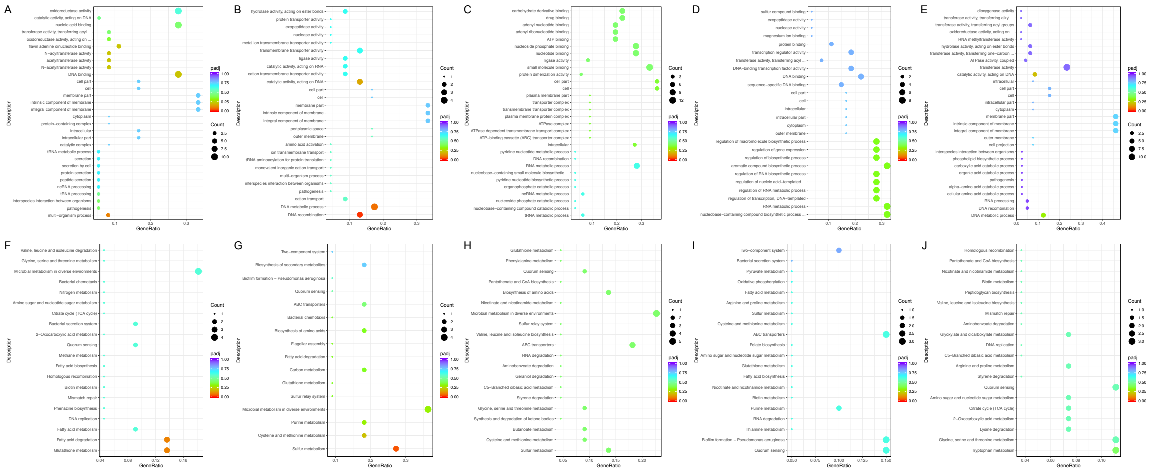

Supplementary Figure 4. Functional enrichment of significantly (padj < 0.05) up-regulated genes in *P. aeruginosa* isolates A2, B7, B11, B29, and B33 compared to the reference strain PAO1. **(A–E)** Enriched GO terms by the significantly up-regulated genes of *P. aeruginosa* isolates A2 **(A)**, B7 **(B)**, B11 **(C)**, B29 **(D),** and B33 **(E)**. **(F–J)** Enriched KEGG terms by the significantly up-regulated genes of *P. aeruginosa* isolates A2 **(F)**, B7 **(G)**, B11 **(H)**, B29 **(I),** and B33 **(J)**.

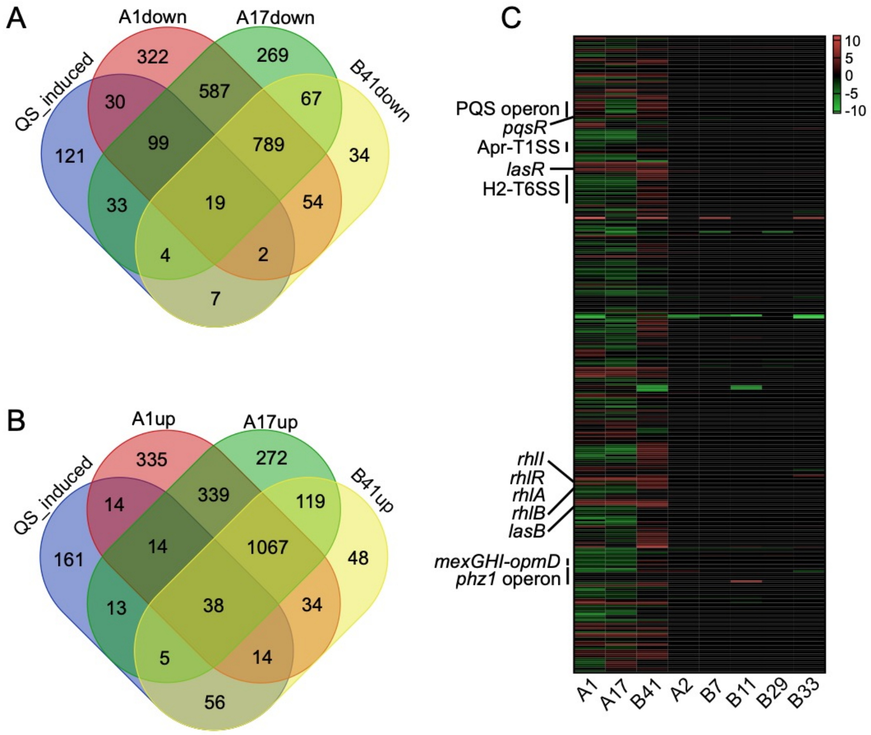

Supplementary Figure 5. Expression levels of QS-induced genes in *P. aeruginosa* clinical isolates compared to PAO1. Numbers of QS-induced genes that significantly (padj < 0.05) down-regulated **(A)** and up-regulated **(B)**. **(C)** Profiling of the 315 QS-induced genes in *P. aeruginosa* clinical isolates. Bar, log2 of fold change.

Supplementary Tables

Supplementary Table 1. Internationally collected *Pseudomonas aeruginosa* strains used for the phylogenetic analysis.

GenBank ID	Sample Name	Genome Size (bp)	Gene Count	Region	Source
CP010555.1	FRD1	6712339	6344	North America	CF isolate
CP008857.1	F30658	7273258	6867	North America	Clinical isolate
CP011317.1	Carb01 63	7497593	7187	Europe	Clinical isolate
CP008856.2	F23197	6535112	6054	North America	Clinical isolate
CP014948.1	N17-1	6370730	5952	Asia	Environmental isolate
NC_011770.1	LESB58	6601757	6026	Europe	CF isolate
NZ_CP006981.1	LESlike7	6467914	5994	North America	CF isolate
NZ_CP006982.1	LES400	6591121	6157	North America	CF isolate
AP012280.1	NCGM2.S1	6764661	6358	Asia	Clinical isolate
AP014651.1	NCGM257	7090694	6777	Asia	Clinical isolate
AP017302.1	IOMTU 133	6897018	6436	Asia	Clinical isolate
CP013144.1	Cu1510	6123018	5836	Asia	Environmental isolate
NC_023019.1	MTB-1	6580038	6186	Asia	Environmental isolate
CP008865.2	S86968	6934277	6530	North America	Clinical isolate
CP008866.2	T38079	6795741	6379	North America	Clinical isolate
CP015117.1	ATCC 27853	6827737	6436	Europe	Clinical isolate
NC_018080.1	DK2	6402658	5960	Europe	CF isolate
CP008867.1	T52373	6322459	5883	North America	Clinical isolate
CP013696.1	12-4-4(59)	6431911	5992	North America	Clinical isolate
NZ_CP007224.1	PA96	6444091	5925	Asia	Clinical isolate
NC_017548.1	M18	6327754	5769	Asia	Plant isolate
CP008861.1	H47921	6836415	6428	North America	Clinical isolate
LN831024.1	NCTC10332	6316979	5922	Europe	Clinical isolate
CP013245.1	VA-134	6400418	5949	North America	Clinical isolate
AP014839.2	8380	6613260	6209	Asia	Clinical isolate
CP008872.2	X78812	6348761	5909	North America	Clinical isolate
CP012066.1	F9676	6368008	5905	Asia	Plant isolate
NZ_CP007147.1	YL84	6433441	5938	Asia	Environmental isolate
CP008859.2	H5708	6334378	5902	North America	Clinical isolate
NC_023149.1	SCV20265	6725183	6380	Europe	CF isolate
NC_021577.1	RP73	6342034	5864	Europe	CF isolate
CP008862.2	M1608	6460023	6060	North America	Clinical isolate
CP008863.1	M37351	6897231	6505	North America	Clinical isolate
NC_008463.1	UCBPP-PA14	6537648	5994	Asia	Clinical isolate

CP015377.1	BAMCPA07-48	7021552	6699	North America	Clinical isolate
NC_020912.1	B136-33	6421010	5904	Asia	Clinical isolate
CP008869.2	W16407	6808844	6434	North America	Clinical isolate
NZ_HG974234.1	PSE305	6762448	6412	Europe	Animal isolate
CP013989.1	USDA-ARS-USMARC-41639	6364583	5904	North America	Animal isolate
SRX4494180	COP2	6557303	6013	Asia	COPD isolate
NC_022360.1	c7447m	6262305	5844	North America	CF isolate
NZ_CP008749.1	PAO1H2O	6264404	5628	North America	Environmental isolate
LN871187.1	PAO1_Orsay	6276469	5826	Europe	Variant of PAO1
NC_022594.1	PAO1-VE13	6265484	5800	North America	Variant of PAO1
AE004091.2	PAO1	6264404	5671	North America	Type strain
NC_022361.1	PAO581	6043974	5662	North America	CF isolate
NC_012560.1	*Azotobacter vinelandii*	5365318	5022		Outgroup

Supplementary Table 2. Information of *P. aeruginosa*-positive patients in this study.

Patient	Gender	Age	Sample	Illness
A1	Male	55	Sputum	COPD, interstitial pneumonia
A2	Male	newborn	Sputum	Acute pneumonia
A11	Female	56	Sputum	COPD, disturbance in respiration for 2 years
A12	Male	79	Sputum	COPD, disturbance in respiration for 5 years
A13	Male	61	Sputum	COPD, pneumonia
A15	Female	69	Sputum	COPD, dyspnea
A16	Male	45	Sputum	COPD, empyema, dyspnea
A17	Female	56	Sputum	COPD, lung infection, dyspnea
A18	Male	87	Sputum	COPD, pneumonia, disturbance in respiration for over 5 years
B2	Female	57	Sputum	COPD, pneumonia, bronchiectasis
B3	Female	64	Sputum	COPD, lung infection, bronchiectasis
B4	Male	55	Sputum	COPD
B6	Male	44	Sputum	COPD, encephalorrhagia
B7	Male	29	Sputum	COPD, spinal injury
B8	Male	59	Sputum	COPD
B9	Male	79	Sputum	COPD, cough, dyspnea
B10	Male	44	Sputum	COPD, encephalorrhagia
B11	Female	78	Sputum	COPD, cough, chest congestion, dyspnea
B13	Male	59	Sputum	COPD
B16	Male	87	Sputum	COPD, acute cough, chest congestion, dyspnea
B17	Female	76	Sputum	COPD, pneumonia, chronic cough, dyspnea
B18	Male	88	Sputum	COPD, cough, dyspnea
B19	Male	69	Sputum	COPD, severe, cough, chest congestion, dyspnea
B20	Male	55	Sputum	COPD, respiratory failure chest congestion, dyspnea
B21	Male	81	Sputum	COPD, chronic cough, dyspnea
B22	Female	59	Sputum	COPD, chronic cough, dyspnea
B23	Female	73	Sputum	COPD, chronic cough, pulmonary embolism
B25	Male	54	Sputum	COPD, chronic cough
B26	Male	59	Sputum	COPD, chronic cough
B28	Male	52	Sputum	COPD
B29	Male	73	Sputum	COPD, chronic cough, dyspnea
B30	Female	28	Sputum	COPD, appendicitis
B31	Male	62	Sputum	COPD
B32	Male	63	Sputum	COPD, lung cancer
B33	Female	33	Sputum	COPD, nasopharyngeal darcinoma
B34	Male	78	Sputum	COPD, chronic cough
B35	Male	55	Sputum	COPD, dyspnea
B37	Male	30	Sputum	COPD, cough, pneumonia, bronchiectasis
B38	Male	86	Sputum	COPD, chronic cough, dyspnea

B39	Male	68	Sputum	COPD, acute cough, lung infection
B40	Male	73	Sputum	COPD
B41	Female	66	Sputum	COPD, chronic cough, dyspnea
B45	Male	68	Sputum	COPD, lung infection

Supplementary Table 3. Mapping information of 22 *P. aeruginosa* clinical isolates to the genome of *P. aeruginosa* PAO1 (NCBI accession number AE004091.2).

Isolates	Avg. depth	Coverage >=1X	Coverage >=4X	Coverage >=10X	Coverage >=20X	Map rate (%)	Mismatch rate (%)	Total base	Total map base
A1	234	95.78	95.73	95.7	95.66	79.01	0.82	9103996500	7192699828
A2	256	97.06	96.96	96.94	96.91	94.71	0.74	1684796700	1595653698
A11	216	96.32	96.27	96.24	96.21	94.12	0.66	1430630100	1346579302
A15	178	96.45	96.41	96.39	96.35	81.48	0.67	1361378400	1109294603
A17	217	96.32	96.27	96.25	96.21	94.33	0.71	1434422700	1353091448
A18	237	95.32	95.21	95.18	95.15	88.4	1.19	1669272600	1475568340
B3	238	96.94	96.9	96.88	96.85	91.19	0.85	7989916200	7286228678
B6	363	96.43	96.37	96.35	96.33	94.72	0.66	2391666300	2265470642
B7	225	96.61	96.55	96.52	96.5	92.14	0.7	1523336400	1403533017
B10	258	96.55	96.36	96.34	96.31	94.81	0.66	1696529700	1608462646
B11	220	96.08	96.01	95.99	95.95	94.62	0.7	1450785900	1372779150
B13	266	96.4	96.36	96.34	96.31	94.15	0.7	1764997500	1661687199
B16	282	95.26	95.21	95.19	95.17	88.09	1.15	1992860700	1755440066
B18	241	96.6	96.54	96.52	96.5	92.55	0.73	1627610400	1506303902
B20	193	96.6	96.56	96.52	96.48	89.88	0.76	1341476700	1205775263
B22	219	97.35	97.32	97.31	97.29	90.59	0.69	1506859800	1364989657
B29	192	96.17	96.11	96.08	96.04	93.17	0.66	1286067900	1198281071
B32	227	96.95	96.91	96.89	96.87	91.7	0.75	1543438200	1415283366
B33	244	96.18	96	95.97	95.94	89.69	1.21	1687006800	1513066429
B34	256	95.71	95.68	95.66	95.62	93.31	0.79	1708387200	1594175827
B35	205	96.62	96.56	96.52	96.48	89.8	0.76	1423474500	1278275455
B41	207	96.46	96.4	96.36	96.31	86.49	0.78	1495372800	1293344561

Supplementary Table 4. Summary of variant sites in 22 *Pseudomonas aeruginosa* clinical isolates compared to the genome of *P. aeruginosa* PAO1.

Isolates	Number of SNPs				Number of InDels			
	Nonsynonymous	Synonymous	Intergenic	Total	Non_shift	Shift	Intergenic	Total
A1	5,687	20,701	3,995	30,383	65	50	345	460
A2	5,774	20,009	4,067	29,850	60	48	380	488
A11	5,238	18,158	3,771	27,167	56	45	321	422
A15	5,471	18,526	3,833	27,830	62	49	325	436
A17	5,236	18,188	3,772	27,196	54	42	321	417
A18	9,416	36,851	7,176	53,443	84	60	573	717
B3	5,299	18,780	3,692	27,771	54	39	341	434
B6	5,199	18,065	3,757	27,021	59	45	322	426
B7	5,558	19,784	4,086	29,428	59	53	350	462
B10	5,173	18,078	3,741	26,992	57	44	320	421
B11	5,684	20,356	3,996	30,036	63	42	341	446
B13	5,223	18,160	3,777	27,160	58	43	319	420
B16	9,429	36,863	7,189	53,481	83	58	575	716
B18	5,327	18,350	3,797	27,474	58	38	360	456
B20	5,832	21,021	4,049	30,902	65	41	379	485
B22	5,464	18,291	3,746	27,501	61	53	347	461
B29	5,161	18,033	3,714	26,908	56	44	321	421
B32	6,089	21,268	4,180	31,537	63	44	358	465
B33	9,294	37,435	7,165	53,894	96	64	581	741
B34	5,712	20,704	4,013	30,429	64	44	342	450
B35	5,828	21,034	4,078	30,940	64	41	382	487
B41	5,788	20,808	4,061	30,657	49	46	369	464

Supplementary Table 5. Numbers of variant genes in 21 *P. aeruginosa* clinical isolates compared with *P. aeruginosa* B29.

	No. of genes with nonsynonymous SNPs			No. of genes with InDels		
Isolates*	Unique	Common	B29 unique	Unique	Common	B29 unique
B13	29	2436	18	7	89	6
B6	30	2431	23	9	89	6
B10	32	2423	31	7	89	6
A11	37	2483	16	5	90	5
A17	38	2433	21	4	87	8
B3	639	1855	610	28	62	33
B18	669	1837	617	26	63	32
A15	674	1886	568	39	66	29
B41	699	1840	614	30	61	34
B20	717	1901	553	32	68	27
A2	718	1867	587	35	65	30
B35	718	1902	552	31	68	27
B7	733	1824	630	44	63	32
B22	734	1835	619	44	63	32
B34	740	1866	588	40	63	32
A1	742	1865	589	45	65	30
B11	768	1866	588	39	58	37
B32	790	1874	580	42	61	34
B33	1290	2065	389	88	61	34
A18	1307	2038	416	84	54	41
B16	1314	2036	418	82	54	41

* Different colors indicate the grouping of these clinical isolates based on the numbers of variant genes (uniquely). Blue, isolates with fewer variant genes. Green, isolates with moderate numbers of variant genes. Orange, isolates with more variant genes.

Supplementary Table 6. Categories of genes with high impact variant sites in 22 *P. aeruginosa* clinical isolates.

Name	Gene ID	Description	No. of strains		
			High impact SNPs	InDels	Total
Protein secretion					
ppkA	PA0074	serine/threonine protein kinase	0	1	1
icmF1	PA0081	type VI secretion protein	0	9	9
fha1	PA0081	Fha domain-containing protein	0	21	21
tli5b1	PA5086	type VI secretion lipase immunity protein	6	7	13
PA0683	PA0683	type II secretion system protein	0	22	22
xcpW	PA3098	type II secretion system protein J	0	2	2
pscP	PA1695	translocation protein in type III secretion	0	19	19
pcrH	PA1707	regulatory protein PcrH	0	22	22
pscK	PA1724	type III export protein	0	22	22
Transport					
tonB2	PA0197	transporter TonB	0	4	4
PA0240	PA0240	porin	0	18	18
PA0241	PA0241	major facilitator superfamily transporter	0	14	14
PA0246	PA0246	major facilitator superfamily transporter	0	1	1
PA0334	PA0334	major facilitator superfamily transporter	0	22	22
oprM	PA0427	outer membrane protein	0	1	1
oprD	PA0958	porin D	1	0	1
PA1025	PA1025	porin	0	2	2
PA1113	PA1113	ABC transporter ATP-binding protein/permease	0	1	1
PA1342	PA1342	ABC transporter	2	0	2
PA1425	PA1425	ABC transporter ATP-binding protein	0	1	1
PA1496	PA1496	potassium channel	1	0	1
PA1507	PA1507	transporter	0	1	1
PA2059	PA2059	ABC transporter permease	0	1	1
PA3039	PA3039	transporter	0	2	2
PA3264	PA3264	transporter	0	7	7
hasE	PA3405	metalloprotease secretion protein	0	1	1
hasD	PA3406	transporter HasD	1	22	23
PA3494	PA3494	electron transport complex subunit E	0	22	22
PA3512	PA3512	ABC transporter permease	0	1	1
PA3889	PA3889	ABC transporter	0	1	1
fecA	PA3901	Fe(III) dicitrate transporter	0	1	1
PA4194	PA4194	ABC transporter permease	0	1	1
PA4502	PA4502	ABC transporter	0	2	2
PA5282	PA5282	major facilitator superfamily transporter	1	14	15
tonB1	PA5531	transporter TonB	1	18	19

mexT	PA2492	transcriptional regulator MexT	0	22	22
QS-induced					
osmC	PA0059	osmotically inducible protein	1	0	1
PA0144	PA0144	hypothetical protein	0	1	1
PA1221	PA1221	hypothetical protein	0	6	6
aprD	PA1246	alkaline protease secretion ATP-binding protein	0	1	1
lasR	PA1430	transcriptional regulator	0	2	2
PA1669	PA1669	hypothetical protein	0	2	2
PA2151	PA2151	alpha-1,4-glucan:maltose-1-phosphate maltosyltransferase	0	1	1
PA2157	PA2157	hypothetical protein	0	2	2
PA2164	PA2164	glycosyl hydrolase	3	0	3
PA2173	PA2173	hypothetical protein	0	1	1
ambE	PA2302	protein AmbE	0	22	22
PA2423	PA2423	hypothetical protein	1	0	1
glyA2	PA2444	serine hydroxymethyltransferase	0	1	1
PA2573	PA2573	chemotaxis transducer	0	2	2
PA2939	PA2939	aminopeptidase	0	1	1
snr1	PA3032	cytochrome C	0	1	1
PA3311	PA3311	signaling protein	0	6	6
ldh	PA3418	leucine dehydrogenase	0	2	2
rhlR	PA3477	transcriptional regulator	0	1	1
osmC	PA0059	osmotically inducible protein	1	0	1
PA0144	PA0144	hypothetical protein	0	1	1
PA1221	PA1221	hypothetical protein	0	6	6
aprD	PA1246	alkaline protease secretion ATP-binding protein	0	1	1
lasR	PA1430	transcriptional regulator	0	2	2
PA1669	PA1669	hypothetical protein	0	2	2
QS-inhibited					
PA0434	PA0434	hypothetical protein	0	6	6
PA1559	PA1559	pseudo	0	22	22
PA2338	PA2338	maltose/mannitol ABC transporter substrate-binding protein	0	1	1
lldP	PA4770	L-lactate permease	0	16	16
Motility					
pilJ	PA0411	twitching motility protein PilJ	0	2	2
fleR	PA1099	two-component response regulator	0	2	2
fimV	PA3115	motility protein FimV	1	5	6
pilA	PA4525	type 4 fimbrial protein PilA	0	1	1
pilB	PA4526	type 4 fimbrial biogenesis protein PilB	0	1	1
pilY1	PA4554	type 4 fimbrial biogenesis protein PilY1	0	1	1

motB	PA4953	flagellar motor protein MotB	0	1	1
pilQ	PA5040	type 4 fimbrial biogenesis outer membrane protein	1	1	2
pilO	PA5042	type 4 fimbrial biogenesis protein PilO	0	2	2

Supplementary Datasets

Supplementary Dataset 1. SNP sites information of 22 *Pseudomonas aeruginosa* clinical isolates compared to the genome of *P. aeruginosa* PAO1 (NCBI accession AE004091.2).

Supplementary Dataset 2. InDel sites information of 22 *P. aeruginosa* clinical isolates compared to the genome of *P. aeruginosa* PAO1 (NCBI accession AE004091.2).

Supplementary Dataset 3. Effect of SNP sites on the function of genes in 22 *P. aeruginosa* clinical isolates.

Supplementary Dataset 4. Significantly enriched KEGG and protein classification terms by the genes with nonsynonymous SNPs in 22 *P. aeruginosa* clinical isolates ($p < 0.05$).

Supplementary Dataset 5. Significantly enriched KEGG and protein classification terms by the genes with shift InDels in 22 *P. aeruginosa* clinical isolates ($p < 0.05$).

Supplementary Dataset 6. Significantly differentially expressed genes of 22 *P. aeruginosa* clinical isolates compared with *P. aeruginosa* PAO1.

Supplementary Dataset 7. Functional enrichment of commonly differentially expressed genes among *P. aeruginosa* clinical isolates A1, A17, and B41.

Supplementary Dataset 8. Functional enrichment of commonly differentially expressed genes among *P. aeruginosa* clinical isolates A2, B7, B11, B29, and B33.

Supplementary Dataset 9. Expression of 315 QS-induced genes in *P. aeruginosa* clinical isolates as determined by RNA-seq.
